# Protective effects of Huangqin Decoction against ulcerative colitis and associated cancer in mice

**DOI:** 10.18632/oncotarget.11426

**Published:** 2016-08-19

**Authors:** Gang Chen, Yang Yang, Chunping Hu, Xiaolan Cheng, Yuehua Xu, Xueting Cai, Min Wang, Chung S. Yang, Peng Cao

**Affiliations:** ^1^ Affiliated Hospital of Integrated Traditional Chinese and Western Medicine, Nanjing University of Chinese Medicine, Nanjing, 210028, Jiangsu, China; ^2^ Laboratory of Cellular and Molecular Biology, Jiangsu Province Academy of Traditional Chinese Medicine, Nanjing, 210028, Jiangsu, China; ^3^ School of Life Science and Technology, China Pharmaceutical University, Nanjing, 210009, Jiangsu, China; ^4^ Department of Chemical Biology, Ernest Mario School of Pharmacy, Rutgers, The State University of New Jersey, Piscataway, NJ, 08854, USA

**Keywords:** Huangqin Decoction, ulcerative colitis, colitis-associated cancer, anti-inflammation and antioxidant

## Abstract

Individuals with ulcerative colitis (UC) are at a high risk for developing colorectal cancer (CRC). Huangqin Decoction (HQD), a traditional Chinese medicinal formula chronicled in the *Shang Han Lun*, is commonly used to treat gastrointestinal symptoms. However, experimental evidence for supporting the clinical practice is lacking. This study used modern biomedical approaches to investigate the protective/preventive effects of HQD in dextran sulfate sodium (DSS)-induced acute/chronic UC and azoxymethane (AOM)/DSS-induced CRC in mice. HQDs were prepared in 4 different ways: HQD-1 and HQD-2 were prepared in boiling water, whereas HQD-3 and HQD-4 were prepared in heated ethanol (70%). For HQD-1 and HQD-3, the 4 constituent herbs were processed together, whereas for HQD-2 and HQD4, these herbs were processed individually and then combined. The mice were administered 9.1 g/kg HQD via oral gavage daily. HQD-1 significantly inhibited DSS-induced acute UC, whereas HQD-3 and HQD-4 exhibited mild ameliorative effects; but HQD-2 had no protective effect and resulted in a higher mortality rate. This higher mortality rate may be due to the greater abundance of baicalein and wogonin in HQD-2 than HQD-1. Furthermore, HQD-1 protected against DSS-induced chronic UC and significantly inhibited AOM/DSS-induced CRC in mice. HQD-1 also inhibited the production of inflammatory cytokines and increased antioxidant capacity both in chronic DSS and AOM/DSS treated mice. Overall, HQD-1 inhibits the development of acute/chronic colitis and prevents colitis-associated CRC, possibly by inhibiting inflammation and preventing oxidative stress induced cellular damage.

## INTRODUCTION

Colorectal cancer (CRC) represents almost 10% of the global cancer incidence burden and is the fourth most common cause of death from cancer worldwide, with an estimated 694,000 deaths in 2012 [1]. Evidence is accumulating that inflammatory bowel disease (IBD) is strongly associated with CRC [2]. Ulcerative colitis (UC) and Crohn's disease (CD) are the two main forms of IBD in humans. In IBD, inflammatory cytokines contribute to the formation of a tumor-supportive microenvironment [3]. In addition, the production of large amounts of reactive oxygen species (ROS) in macrophages, neutrophils, and other inflammatory cells in inflamed tissues results in oxidative stress and impairment of antioxidant defenses. Increased ROS levels lead to protein damage, lipid peroxidation, and DNA damage, all of which may lead to genetic and epigenetic alterations, which drive the development of carcinoma [4, 5]. The development of CRC from IBD is a long process and there are opportunities for intervention to prevent the development of this disease [6, 7].

Aminosalicylates have been used extensively to treat patients with IBD. Several studies have demonstrated the preventive effects of aminosalicylates against colitis-associated CRC; however, such an effect was not demonstrated in other studies [8]. Corticosteroids have been used widely to treat patients with IBD and have been found to prevent CRC. Nonetheless, adverse effects during long-term use of corticosteroids are a concern [9]. Treatment with biologics is a novel approach for controlling IBD. However, data regarding the preventive effects of biologics on CRC development in IBD patients are still lacking [10]. Therefore, new drugs and approaches for treating IBD patients and preventing IBD-associated CRC are urgently needed.

Huangqin Decoction (HQD), a traditional Chinese formula from the *Shang Han Lun*, is made from four herbs: *Scutellaria baicalensis* Georgi, *Paeonia lactiflora* Pall, *Glycyrrhiza uralensis* Fisch, and *Ziziphus jujuba* Mill (Supplementary Table S1) [11]. HQD is commonly used to treat gastrointestinal symptoms including diarrhea, nausea, and vomiting [12]. Modern research has demonstrated that HQD exhibits significant anti-inflammatory effects in mice with UC [11]. PHY906, a preparation derived from HQD, has been shown to enhance the anti-tumor activity of CPT-11 and decrease the weight loss caused by CPT-11 [13, 14]. Baicalin, the principle bioactive constituent of *Scutellaria baicalensis* Georgi, has a strong antioxidant activity and an anti-colitis effect [15–17]. However, the protective/preventive effects of HQD against acute UC, chronic UC, and UC-associated CRC have not been systematically studied.

In the present study, we investigated the protective effect of HQD and the underlying mechanisms in mouse models of dextran sulfate sodium (DSS)-induced acute and chronic UC and UC-associated cancer. The effects of the different ways of preparation of HQDs on their biological activities were also studied.

## RESULTS

### Protective effects of differently prepared HQDs against DSS-induced acute UC in mice

**Figure 1:**
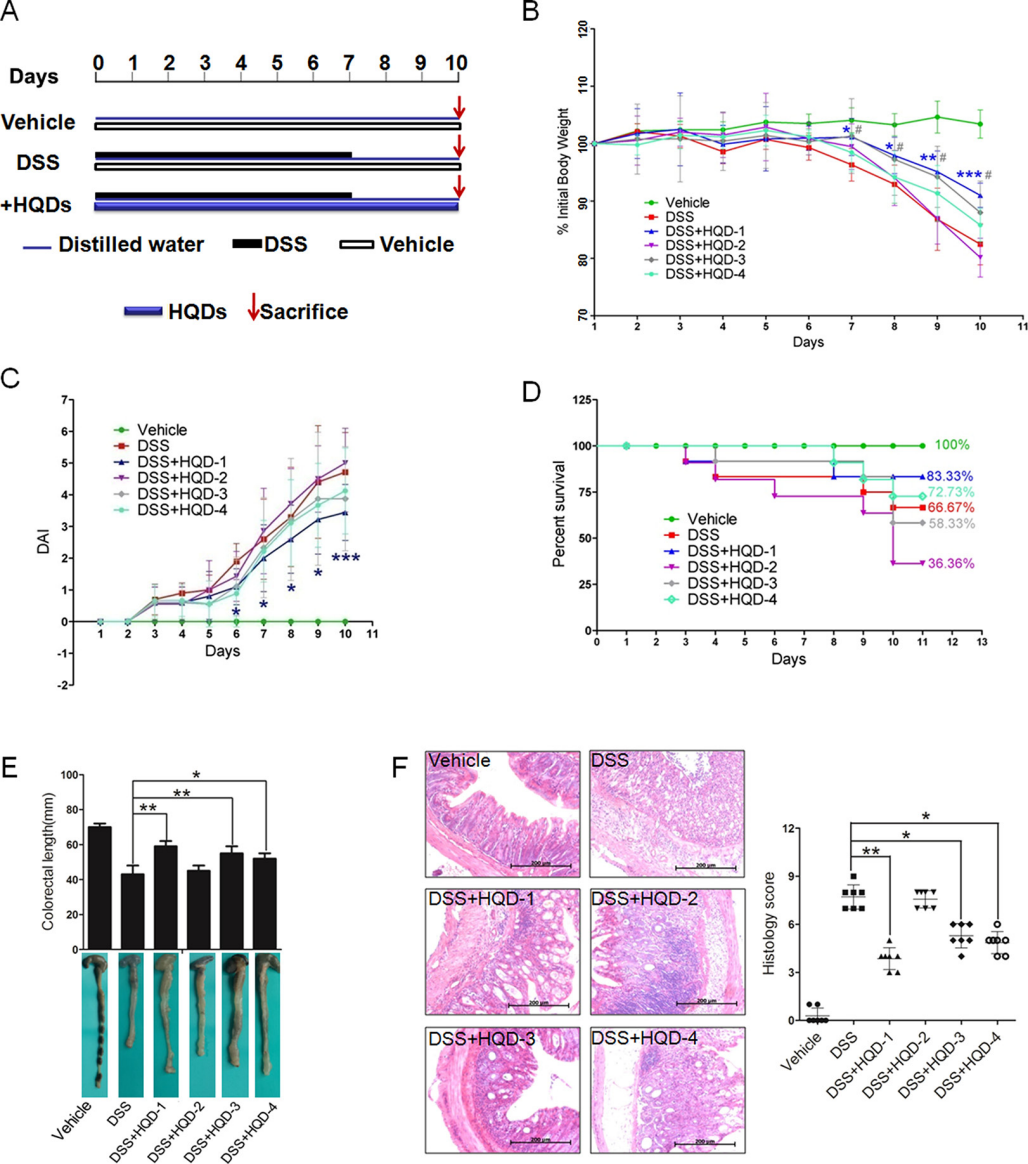
Protective activity of HQD preparations against DSS-induced acute ulcerative colitis in mice (**A**) Animal model of acute DSS-induced UC in C57/BL6 mice. (**B**) Body weight changes of the mice. (**C**) Disease activity index (DAI). (**D**) Survival curves. (**E**) Photographs of the colon lengths. (**F**) Representative H&E-stained colon sections (magnification ×100) and histology score. Data are presented as mean ± SD of 7 mice in each group.*P < 0.05; **P < 0.01; ***P < 0.001, HQD-1 versus DSS-treated group.^#^*P* < 0.05; ^##^*P* < 0.01; ^###^*P* < 0.001, HQD-2 versus DSS-treated group.HQD, huangqin decoction.

Treatment of mice with 3% DSS in drinking water for 7 days induced colitis symptoms characterized by weight loss, loose stool and hematochezia. The mice in the vehicle group exhibited a normal growth rate. In the DSS group, the body weight of the mice did not increase during the first four days and significant decreased starting on the fifth day. The mice in the DSS+HQD-1 group exhibited less weight loss in comparison with mice in the DSS group. HQD-3 and HQD-4 also partially prevented body weight loss. However, HQD-2 did not prevent DSS-induced body weight loss (Figure 1B) and elevated the DAI (Figure 1C).

The survival rates of the groups of mice treated with HQD-1 (83.33%) and HQD-4 (72.73%) were higher in comparison with that of the DSS group (66.67%). However, the survival rates of the DSS+HQD-2 (36.56%) and DSS+HQD-3 (58.33%) groups were lower than that of the DSS group (Figure 1D). Colon shortening is a visual index that reflects the severity of colorectal inflammation [18]. The colorectal length of the group of mice treated with DSS was shorter than that of the vehicle-treated group. Administration of HQD-1, HQD-3, and HQD-4 significantly inhibited colon shortening, and HQD-1 was the most effective preparation. However, HQD-2 did not significant inhibit colorectal shortening (Figure 1E). Severe epithelial injury, distortion of crypts, and inflammatory cells infiltration in the mucosa and submucosa appeared in the colorectal tissues of DSS-treated mice as examined by H&E staining and histopathological evaluation. HQD-1 administration preserved colorectal crypt structure and reduced the severity of inflammation. Treatment with HQD-3 or HQD-4 also decreased the histological scores. However, HQD-2 did not ameliorate pathological damage (Figure 1F).

The anti-inflammatory activity of each HQD preparation was further assessed by qRT-PCR. Expression levels of transcripts encoding TNF-α, IL-1β, IL-6, MCP-1, and CSF-1, and COX-2 were significantly elevated after DSS challenge. HQD-1 inhibited the expression of all five assessed cytokines and COX-2 after DSS challenge; whereas HQD-2, HQD-3, and HQD-4 were less effective and only reduced the expression levels of some of the assessed cytokines (Figure S1).

These results indicate that HQD-1 strongly inhibited DSS-induced colitis. HQD-3 and HQD-4 exhibited a mild ameliorative effect, but HQD-2 had no protective effect and caused a higher mortality rate in DSS-treated mice.

### HPLC analysis and the effects of HQD constituents on DSS-induced acute UC in mice

**Figure 2:**
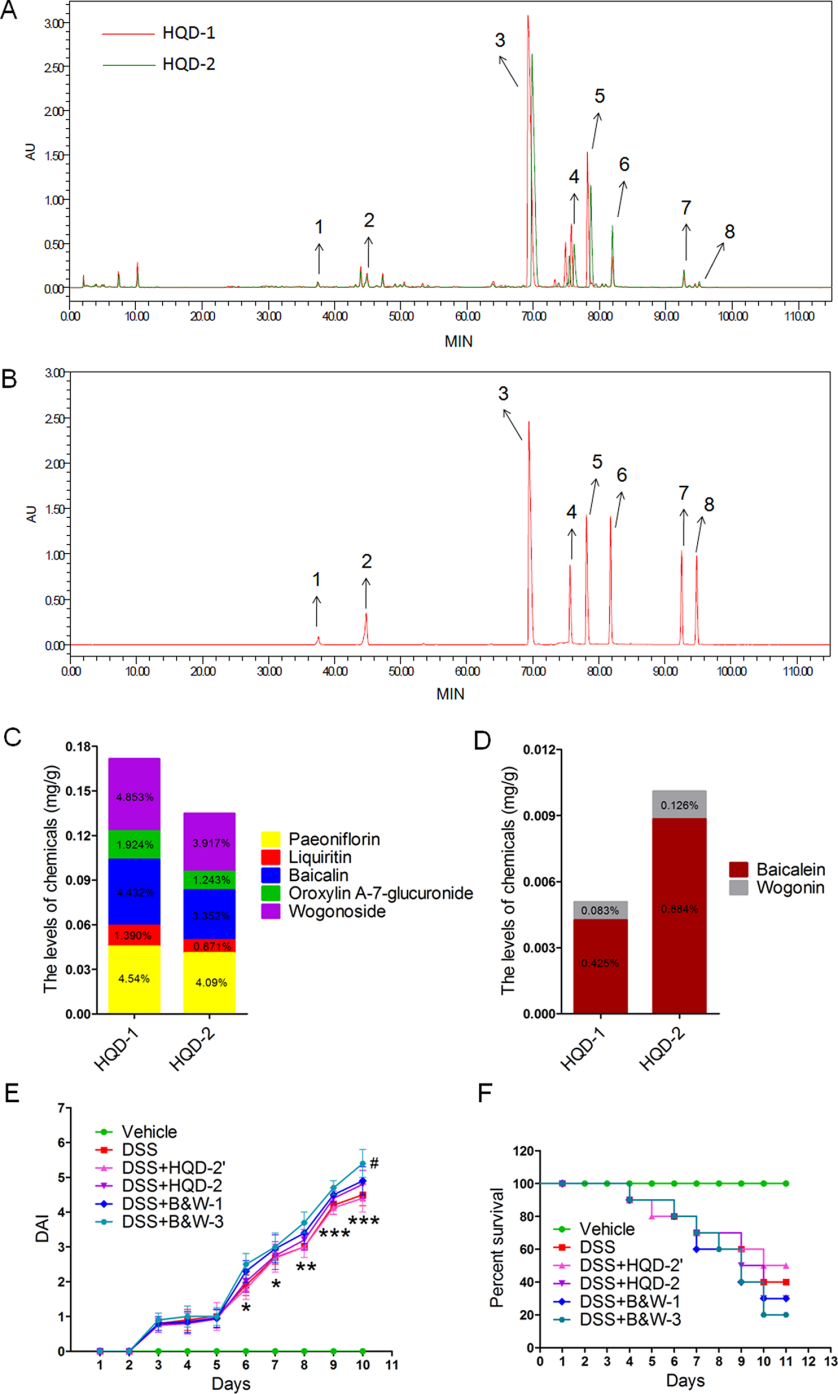
HPLC chromatogram of HQD-1 and HQD-2(A) Reference standards (**B**). The content of several chemical constituents in HQD-1 (**C**) and HQD-2 (**D**). Disease activity index (DAI) (**E**) and survival curves (**F**).HQD, huangqin decoction.

In order to determine the molecular basis for the high death rate in the HQD-2-treated group, we analyzed the differences in the chemical compositions of HQD-1 and HQD-2 by HPLC. Chromatograms of the constituents are shown for HQD-1 and HQD-2 (Figure 2A) and for single herbs (Figure S2). The 8 main peaks in the chromatograms were identified by comparison with reference standards (Figure 2B, Figure S3, Table S2) [12, 19]. We found no pronounced differences between the constituent profiles of HQD-1 and HQD-2; however, the areas under peaks 3, 4, 5, 6, and 7 differed (Figure 2A). The areas under peaks 3, 4, and 5, corresponding to baicalin, oroxylin A-7-glucuronide, and wogonoside, respectively, were larger in HQD-1 than in HQD-2. However, the areas under peaks 6 and 7, corresponding to baicalein and wogonin, respectively, were larger in HQD-2 than in HQD-1. These results suggest that differences in the amounts of these chemical constituents lead to the observed differences in the protective and toxic effects of HQD-1 and HQD-2. The results of the quantitative analyses of the key constituents in HQD-1 and HQD-2 are shown in Figure 2C and 2D. The validation of this quantitative HPLC method showed that the method was sensitive, precise, and stable (Table S4).

If the toxic effect of HQD-2 was due to the deleterious effects of baicalein and wogonin, would decreasing the dose of HQD-2 by 50% (designated as treatment HQD-2′) be beneficial? We found that HQD-2′ was less toxic than that of HQD-2; however, the UC symptoms of the HQD-2′-treated group were similar to those of the DSS group (Figure 2E–2F and Figure S4). We also examined the effects of the combination of purified baicalein and wogonin at the same (referred to as B&W-1) and triple (B&W-3) the concentrations present in HQD-2. As shown in Figure 2E, 2F, and S3, B&W-1 and B&W-3 worsened colitis symptoms. Furthermore, B&W-3 increased the severity of inflammation. These results suggest that the toxicity of HQD-2 is due to the higher contents of baicalein and wogonin. The lower contents of baicalin, oroxylin A-7-glucuronide, and wogonoside in HQD-2 may be the reason for its lower effectiveness in protection against UC.

### Protective effect of HQD-1 against DSS-induced chronic UC in mice

**Figure 3:**
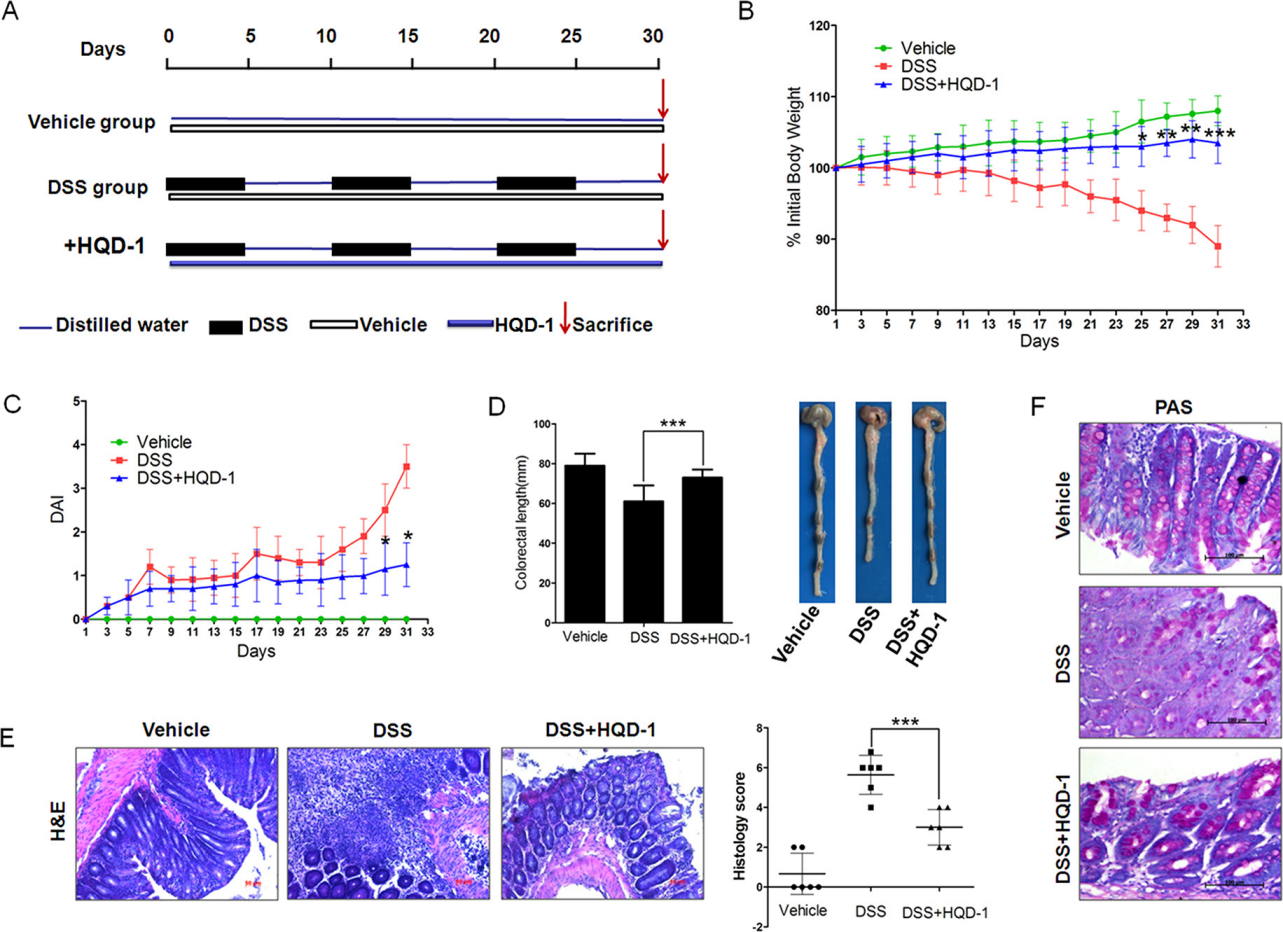
HQD-1treatment ameliorated DSS-induced chronic ulcerative colitis in mice (**A**) Animal model of chronic DSS-induced UC in C57/BL6 mice. (**B**) Body weight changes after DSS induction of colitis. (**C**) DAI. (**D**) Statistics of colon length of each group.(**E**) Representative H&E-stained colon sections (magnification×100) and histology score. (**F**) Periodic acid-Schiff (PAS) staining (magnification ×400). Data are presented as mean ± SD of 6 mice in each group.*P < 0.05; **P < 0.01; ***P < 0.001, versus DSS-treated group. HQD, huangqin decoction.

Because of its strong protective effect against acute UC, we studied the protective effect of HQD-1 against chronic UC. DSS inhibited the normal growth and caused a decrease in body weight; HQD-1 administration significantly ameliorated weight loss (Figure 3B). Consistent with the change in body weight, the DSS+HQD-1 group had a DAI significantly lower than that of the DSS group (Figure 3C). Colon length shortening by DSS was also prevented by HQD-1 (Figure 3D). Histopathological evaluations were performed on H&E-stained colon sections to evaluate the anti-inflammatory activity of HQD-1 (Figure 3E). The DSS-treated mice displayed severe epithelial injury and inflammatory cell infiltration in the mucosa and submucosa, which was significantly reduced by HQD-1. To further evaluate epithelial cell damage, periodic acid-Schiff (PAS) staining for mucins produced by goblet cells in the colon was performed [20]. The DSS group showed a significant decrease in the number of PAS-positive cells in colorectal tissues in comparison with that of the mice in the vehicle group. HQD-1 treatment prevented the decrease in the number of PAS-positive cells in DSS-treated mice (Figure 3F).

### Effects of HQD-1 on the production of inflammatory cytokines and oxidative stress in DSS-induced chronic colitis mice

**Figure 4:**
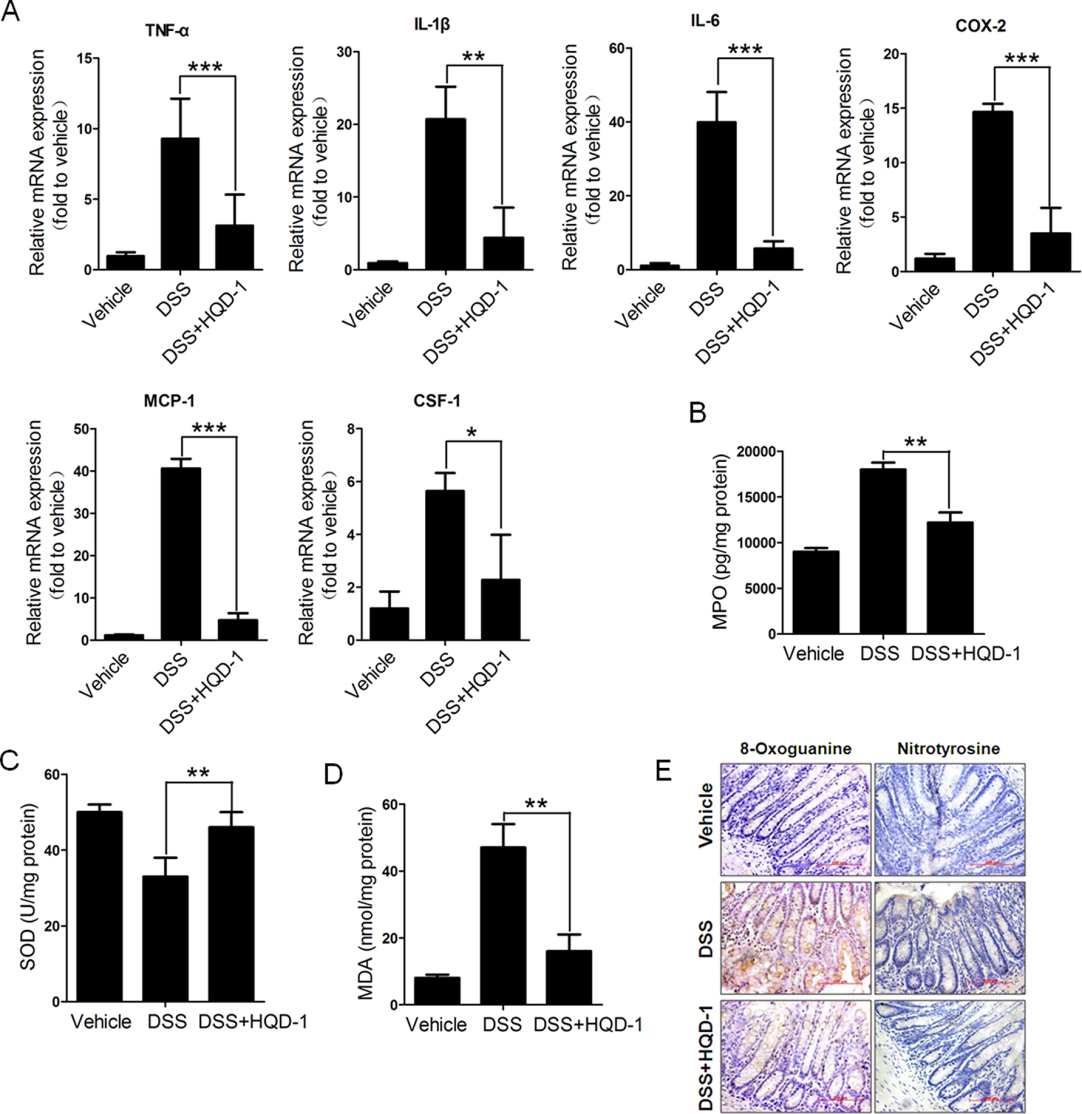
The effects of HQD-1 on the production of inflammatory cytokines and oxidative stress in colons during chronic ulcerative colitis induced by DSS in mice (**A**) Quantitative RT-PCR analysis for TNF-α, IL-1β, IL-6, COX-2, MCP-1 and CSF-1 was performed on total RNAs extracted from the colons. (**B**) MPO, (**C**) SOD activity, (**D**) MDA and (**E**) levels of 8-Oxoguanine and nitrotyrosine (magnification ×400).*P < 0.05; **P < 0.01; ***P < 0.001, versus DSS-treated group. HQD, huangqin decoction.

Colorectal mRNA levels of inflammatory cytokines TNF-α, IL-1β, IL-6, MCP-1, and CSF-1, and COX-2, were measured in mice with chronic colitis. Substantially increased mRNA levels of the assessed cytokines and COX-2 were found in the colorectal tissue of the DSS group; these mRNA levels were decreased by HQD-1 treatment (Figure 4A). The mice subjected to a 2% DSS challenge also showed a significantly increased MPO level in comparison with the mice in the vehicle and DSS + HQD-1 group (Figure 4B).

Oxidative stress is involved in the pathogenesis of colitis. As a major part of the antioxidant defence system, superoxide dismutase (SOD) is an important superoxide radical scavenger [21]. The activity of SOD was decreased after DSS treatment, while HQD-1 prevented SOD activity from decrease (Figure 4C). UC is associated with an increased level of MDA [22, 23]. As expected, MDA content was significantly increased after DSS treatment and the increase was mostly prevented by HQD-1 (Figure 4D). Strong 8-oxoguanine and nitrotyrosine immunostaining was observed in the colorectal tissue of the DSS group; HQD-1 treatment reduced the number of immunostained cells (Figure 4E).

### Preventive effect of HQD-1 on AOM/DSS-induced colon cancer in mice

**Figure 5:**
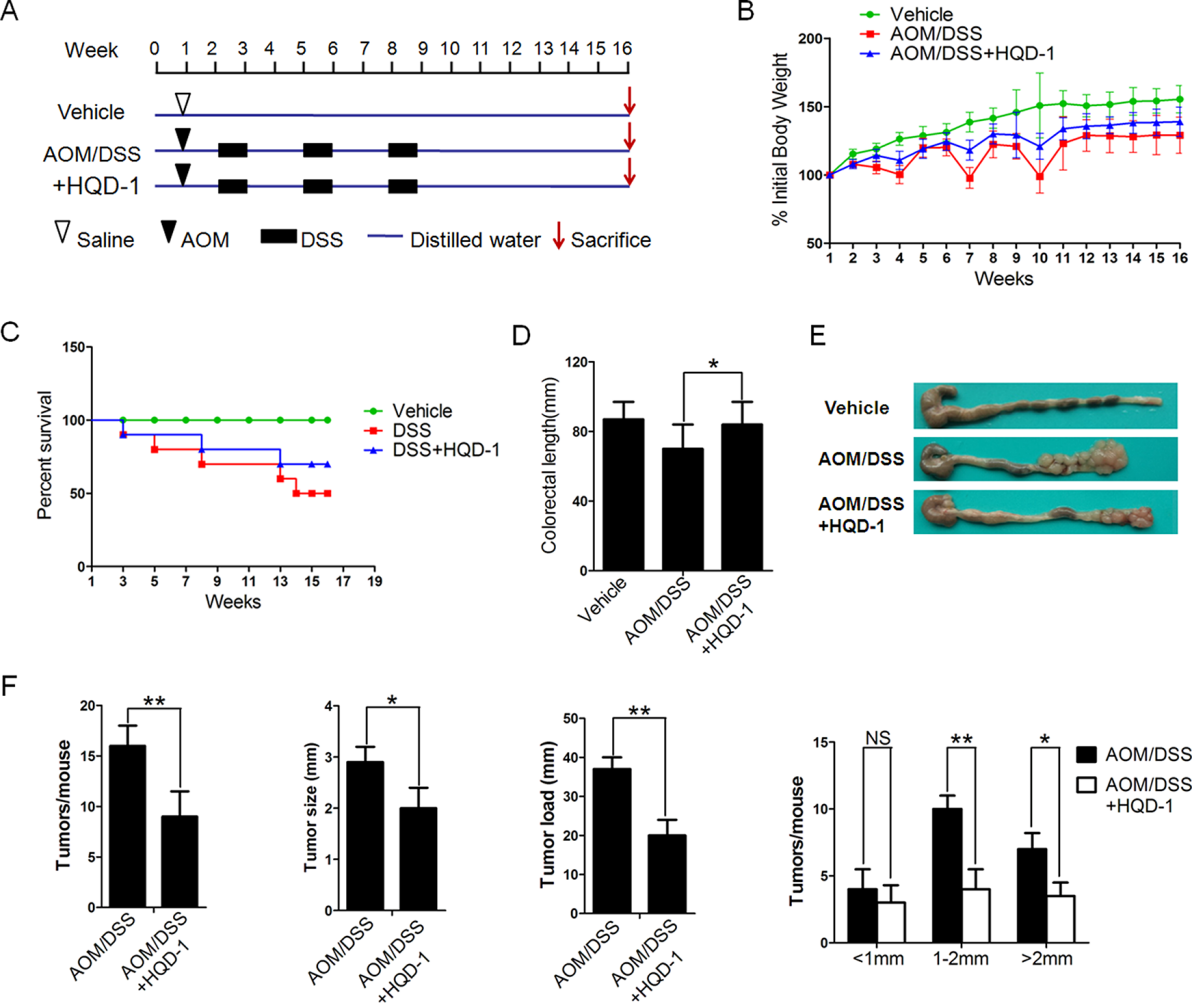
The preventive effects of HQD-1on colitis-associated tumorigenesis induced by AOM/DSS (**A**) Animal model of AOM/DSS-induced CAC in mice. (**B**) Body weight changes after AOM/DSS induction of CAC. (**C**) Survival curves. (**D**) Statistics of colon length of each group. (**E**) Colon morphology. (**F**) Tumor numbers, diameter, tumor load and distribution were measured.*P < 0.05; **P < 0.01; ***P < 0.001, versus DSS-treated group and NS, not significant. HQD, huangqin decoction.

The preventive effect of HQD-1 on AOM/DSS-induced tumorigenesis was assessed. The body weights of mice were reduced substantially after each exposure cycle to DSS and the mice partially regained body weight when DSS was withdrawn. In the HQD-1 group, body weight loss was less extensive than the mice treated with AOM/DSS only (Figure 5B). Survival curves showed that HQD-1 treatment increased the survival rate of the AOM/DSS treated mice (Figure 5C). Moreover, shortening of colon length was prevented by HQD-1 (Figure 5D). Macroscopic images of the colon are shown in Figure 5E. The average number of tumors per mouse, tumor size, average tumor load, and tumors of different sizes in the HQD-1 group were all significantly decreased in the AOM/DSS treated mice (Figure 5F).

Histological examination of tumor tissue and surrounding tissues was performed (Figure S5). As shown by H&E staining, the tumor tissues of the AOM/DSS group displayed colonic adenomas with high-grade dysplasia, whereas the HQD-1-treated mice showed low-grade dysplasia. In comparison with the AOM/DSS group, the group received HQD-1had fewer crypt loss and more normal epithelial cells in tissues surrounding the tumors. PCNA immunochemistry was performed to analyse proliferation of cells in tumors and their surrounding tissues. Extended areas of PCNA-labelled cells were observed in tumor tissues and surrounding tissues in AOM/DSS treated mice; and the PCNA-positive area was substantially lowered by HQD-1 treatment. Furthermore, the apoptosis rate, as measured by TUNEL staining, in tumor tissues from the HQD-1 treatment group was higher in comparison with that of the group received AOM/DSS alone.

### Suppression of inflammatory cytokine production and improvement of anti-oxidative activity by HQD-1 in mice with AOM/DSS-induced CRC

**Figure 6:**
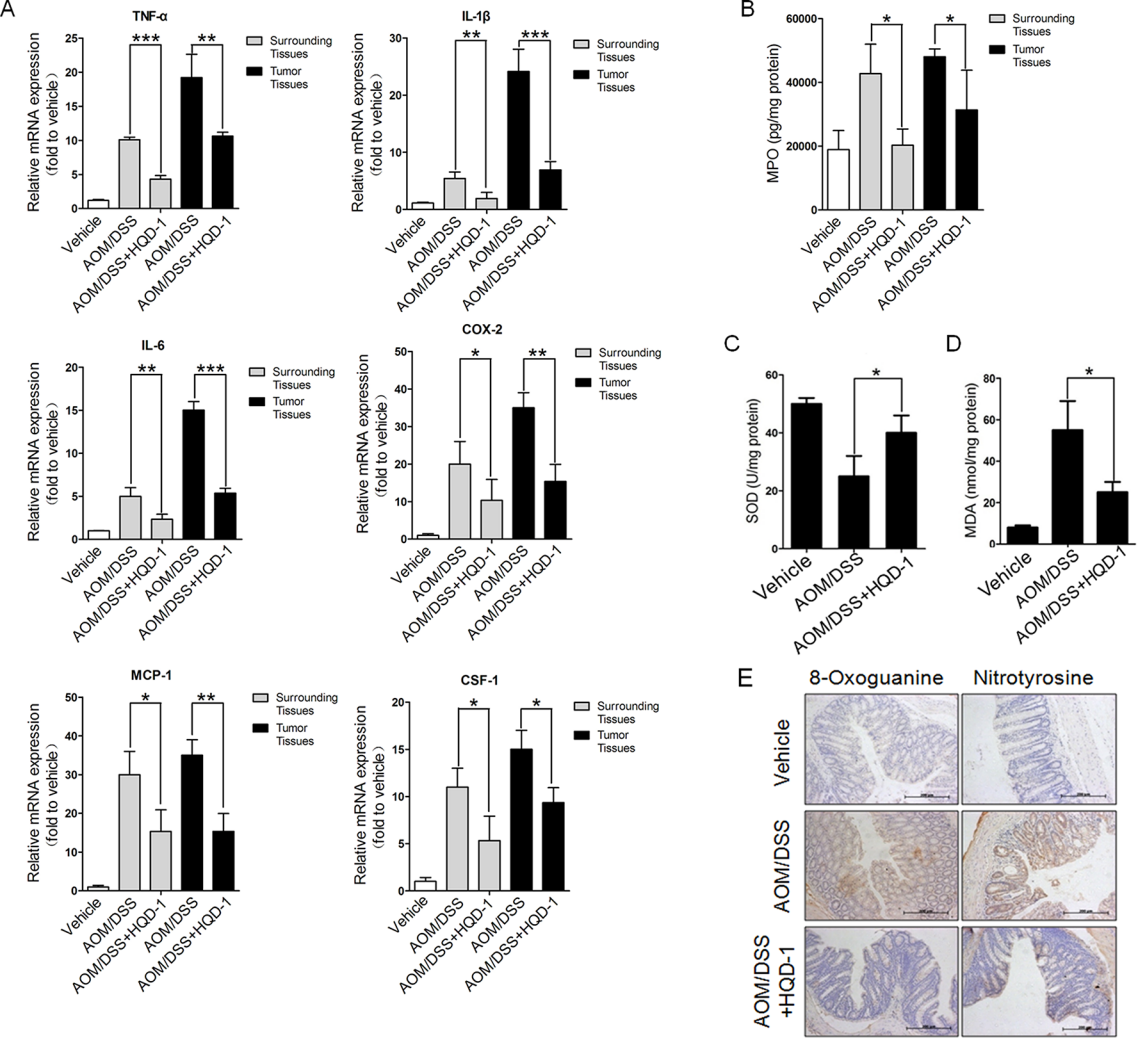
The effects of HQD-1 on the production of inflammatory cytokines and oxidative stress in tumor and surrounding tissues during colitis-associated colon cancer Effects of HQD-1 on the production of inflammatory cytokines (**A**) and MPO (**B**) in colons of AOM/DSS-treated mice. SOD activity (**C**) MDA (**D**) 8-Oxoguanine and nitrotyrosine (**E**) levels in colonic tissues of AOM/DSS-treated mice (magnification ×400).*P < 0.05; **P < 0.01; ***P < 0.001, versus DSS-treated group. HQD, huangqin decoction.

To assess the roles of inflammation and oxidative stress in colitis-associated cancer [4, 24], several markers were measured. The mRNA levels of colorectal inflammatory cytokines TNF-α, IL-1β, IL-6, CSF-1, and MCP-1, and COX-2 were increased in tumor and surrounding tissues from AOM/DSS-treated mice. The mRNA levels of inflammatory cytokines and COX-2 in tumor tissues were higher than those of surrounding tissues. HQD-1 reduced the mRNA levels of these cytokines and COX-2 caused by AOM/DSS treatment (Figure 6A). HQD-1 treatment also significantly decreased MPO levels caused by AOM/DSS-treatment in tumor and surrounding tissues (Figure 6B).

The HQD-1-treated group showed significantly higher SOD activity in tissues surrounding the tumors in comparison with that of the AOM/DSS control group (Figure 6C). The HQD-1-treated group also markedly decreased the levels of MDA, 8-oxoguanine, and nitrotyrosine in the AOM/DSS treated mice (Figure 6D–6E).

## DISCUSSION

IBD can greatly decrease the quality of life because of diarrhea, abdominal pain, and other clinical symptoms. More seriously, patients with IBD have a high risk for CRC [25, 26]. In this study, we demonstrated that HQD, a traditional Chinese medicine commonly used for treatment of gastrointestinal symptoms, has protective or preventive activity against DSS-induced colitis and AOM/DSS-induced CRC in mice. To our knowledge, this is the first systematic and thorough investigation of HQD in the prevention of UC and associated CRC. A scheme summarizing these actions and the underlining mechanisms are shown in Figure S6. A novel result is that the biological activities are different when HQD is prepared by different procedures.

Our results showed that HQD-1 had a strong protective effect against DSS-induced acute UC, whereas HQD-2, had no protective effect and increased mortality rate (Figure 1). The high mortality rate associated with HQD-2 may be due to its higher baicalein and wogonin content (Figure 2). HQD-1 was prepared by decocting the four constitutive herbs together with hot water and showed the best efficacy among the four HQDs, prepared by different methods. Apparently, decocting the four herbs together resulted in lower contents of baicalein and wogonin, compared to decocting the four herbs separately and then combined. The results demonstrated that the traditional decoction method (HQD-1) produced the best product. The interactions among herbs during decoction are interesting topics for further study.

Because of the superior protective effect of HQD-1 against acute colitis, we selected it for further studies. Our results showed that HQD-1 effectively ameliorated chronic UC and prevented colitis-associated cancer. These activities were associated with decreases in markers for oxidative stress (8-oxoguanine, nitrotyrosine, and MDA), neutrophil infiltration (MPO), mRNA levels of inflammatory cytokines and COX-2, as well as a higher rate of cell proliferation and an increased rate of cell apoptosis.

To identify the active constituents and elucidate their mechanisms of action of a herbal medicine is a challenging task. For HQD, the activities of some of the major constituents have already been reported. Baicalin, the principal flavonoid from Huangqin (*Scutellaria baicalensis* Georgi), has been shown to have anti-inflammatory and antioxidant activities. For example, baicalin induced Foxp3 protein expression in cultured T cells, and stimulated Treg cells to decrease Th17 cells in inflammatory tissues [27, 28]. Baicalin also suppresses the expression of pro-inflammatory cytokins as well as the expression of phospho-IKKα/β and phospho-NF-κB p65 [29]. Baicalin could also protect against lead-induced renal oxidative damage in mice [30]. It was demonstrated that baicalin increased the antioxidant capacity via promoting the nuclear translocation of NF-E2-related factor 2 (Nrf2) [31]. The pro-apoptotic activity and possible mechanisms of action of baicalin in cell lines have also been reported [32–35]. Wogonoside, another major component in Huangqin, has been shown to inhibit the activation of NF-kB and NLRP3 inflammasomes in DSS-induced colitis and colitis-associated tumorigenesis in mice [36, 37]. Paeoniflorin, a monoterpene glucoside from the root of Shaoyao (*Paeonia lactifloraI* Pall) is another major ingredient in HQD-1. Paeoniflorin has been reported to suppress the production of inflammatory factors *in vitro* [38, 39] and *in vivo* [40] and significantly reduce the severity of colitis in mice [41]. Liquiritin, an active component of Gancao (*Glycyrrhiza uralensis* Fisch), has been shown to have the properties of inhibiting LPS-stimulated elevation of pro-inflammatory mediators [42] and antioxidant properties in decreasing the levels of MDA, increasing the ratio of GSH/GSSG and the activities of SOD, CAT, and GSH-Px, and decreasing 8-OHdG [43]. In the colitis-associated cancer mouse model, reactive oxygen species and inflammation are intertwined to promote colitis and colon carcinogenesis. The presently observed protective or preventive effect of HQD are likely due to the combined antioxidant and anti-inflammatory actions of these constituents in HQD. The pro-apoptotic activity and suppression of the Wnt/b-catenin [44] or Notch pathway [45] by some of these compounds may also contribute to prevent the development of CRC. These mechanisms collectively would account for most of the inhibitory activity of HQD-1 against acute/chronic UC and colon carcinogenesis in our mouse models. The possible contributions of other constituents remain to be elucidated.

A number of compounds that are used to treat UC have been studied for their potential in prevention of CRC. For example, 5-aminosalicylates (5-ASA) are widely used to treat patients with UC, but their effectiveness in prevention against CRC is still under debate [8, 46]. Glucocorticoids are useful in the treatment of acute flares of IBD; however, they are not suitable for long-term use to prevent cancer because of their adverse effects [8, 29]. HQD, commonly used to treat gastrointestinal disorders, is inexpensive and generally considered to be safe. It may have a higher potential for use in the prevention of CRC, especially in patients with UC.

In the present study, we demonstrate that HQD-1 is effective in protecting against UC and preventing colitis associated-CRC. The results suggest the therapeutic potential of using HQD-1 to treat acute/chronic UC and to prevent associated CRC. Future research in well-designed clinical trials is needed to further assess the efficacy of HQD-1 as well possible side effects.

## MATERIALS AND METHODS

### Reagents

Azoxymethane (AOM) and DSS (MW 36,000–50,000) were obtained from Sigma-Aldrich (St Louis, MO, USA). Maxima^®^ SYBR Green/ROX qPCR Master Mix (2×) and Maxima^®^ First Strand cDNA Synthesis Kit were purchased from Fermentas life science (Waltham, MA, USA). Immunohistochemistry kit and myeloperoxidase (MPO) (m) ELISA Kit were purchased from Boster (Wuhan, Hubei, China.).

### Composition and preparation of HQDs

All medicinal herbs were provided by Jiangsu Province Hospital on Integration of Chinese and Western Medicine (Nanjing, Jiangsu, China). Huangqin Decoction is made from four herbs: *Scutellaria baicalensis* Georgi, *Paeonia lactiflora* Pall, *Glycyrrhiza uralensis* Fisch, and *Ziziphus jujuba* Mill (Table S1). HQDs were prepared in four different ways. For HQD-1, the 4 different herbs were soaked together in 10 volumes of distilled water for 30 min, heated to boiling and maintained at 100°C for 30 min. The decoction was filtered and the residual herbs were added 8 columns of water for a second extraction. The filtrates obtained from the two cycles of extraction were combined. For HQD-2, the 4 different herbs were extracted individually under similar conditions, and then the 4 herbal extracts were combined. HQDs-3 and -4 were prepared with 70% ethanol at 60°C similarly to HQD-1 and -2, respectively.

Waters e2695 Alliance HPLC system (Waters Corp., MA, USA) with 2489 UV/Vis DAD detector was used for qualitative analysis of HQD aqueous extracts. Extracts were separated by an Inertsil ODS-SP C18 column (250 mm × 4.6 mm, 5 μm). The injection volume was 10 μl. The mobile phase consisted of linear gradients of 0.1% (v/v) formic acid (A) and acetonitrile (B): 0–15 min, 100–95%A (v/v), 0–5%B (v/v); 15–30 min, 95–85% A, 5–15% B; 30–60 min, 85–77% A, 15-23% B; 60–90 min, 77–55% A, 23–45% B; 90–110 min, 65–40% A, 45–60% B; 110–115 min, 40–90% A, 60–5% B; 115–120 min, 40–90%A, 60–5%B. The mobile phase flow rate was 1 ml/min. The column was run at 30°C. Purified chemical reference substances (Table S2) were used for quantitation by HPLC [19, 47].

### Animals and experimental protocols

All animal experiments were conducted in accordance with the U.S. NIH Guidelines for the Care and Use of Laboratory Animals. Pathogen-free 8-week old C57BL/6 male mice were allowed to acclimate for 1 week before experiment.

Acute colitis was induced by administering 3% DSS in drinking water for 7 days and followed by switching mice to normal water for 3 days (a total of 10 days) [48]. In HQD (HQD-1, -2, -3, and -4) treated groups, the mice were given 9.1 g/kg HQD via oral gavage daily for all the 10 days. Mice in the vehicle control group and DSS group consumed the same volume of water as controls (Figure 1A).

Chronic colitis was induced in mice by three cycles of “5 days of 2% DSS in drinking water, alternating with 5 days of normal water”, for a total 30 days [49]. In the HQD-1 group, the mice were given 9.1 g/kg HQD-1 daily during all 30 days via oral gavage. Mice in vehicle group and DSS group consumed the same volume of water (Figure 2A).

For CRC studies, mice were injected intraperitoneally at the beginning of week 2 (1 week after the initiation of HQD-1) with AOM in physiological saline at a daily dose of 10 mg/kg. One week later, 2% DSS was given in the drinking water for 7 days, followed by 14 days of regular water. The DSS treatment was repeated for a total of 3 cycles, and the animals were sacrificed at the end of week 16 [50]. In the HQD-1 group, the mice were given 9.1 g/kg HQD-1 daily via oral gavage, starting one week before AOM injection, throughout the experiment. Mice in the vehicle control group and DSS group consumed the same volume of water (Figure 5A).

### Evaluation of colitis

Body weight, hematochezia and stool characteristics were monitored daily. Animals were sacrificed by cervical dislocation under anesthesia. The colon was removed and length was measured. The distal colon was fixed in 4% paraformaldehyde, embedded in paraffin and stained with H&E according to standard protocols. Histological scoring was performed as described [51]. The disease activity index [DAI= (Weight loss score + Stool characteristics score + Hematochezia score)/3] evaluation was performed as described [52].

### Quantitative RT-PCR

Total RNA was extracted from colon tissues using trizol reagent and then RNA was converted to cDNA by reverse transcriptase according to the manufacturer's instruction. Both the tumor in clusters and its surrounding nontumorous tissues (within the distance of 5 mm from tumor) were collected. Primers used for the reactions were purchased from Genscript and the sequences were listed in Table S3. Real-time qPCR analysis for mRNA expression was performed using SYBR Green probes and an ABI 7500. The mRNA expression was normalized against GAPDH expression.

### Enzyme-linked immunosorbent assay

Colon tissues from each group were homogenated with lysis buffer to extract total protein. The homogenate was centrifuged at 12,000 × g at 4°C for 15 min. The amount of total protein was determined by BCA protein assay kit. The amount of myeloperoxidase (MPO) in colorectal tissues was quantified according to the manufacturer's instructions [53].

### Immunohistochemical analyses

Paraffin-embedded sections (4μm) of colon were analysed for 8-oxoguanine [54] and nitrotyrosine [55] and PCNA [56]. After retrieving of antigens, the expression was detected using respective primary antibodies at 4°C overnight. Biotin-labelled secondary antibody and streptavidin-HRP were incubated for 30 min at room temperature. Immunoreactions were detected using 3-3-diaminobenzidine followed by counterstaining with haematoxylin.

### Statistical analysis

Statistical analysis was performed using SPSS software version 15.0. Data are presented as mean ± SD. Unpaired Student *t* tests were used to compare the means of two groups. *P* value < 0.05 was considered to be statistically significant.

## SUPPLEMENTARY MATERIALS FIGURES AND TABLES



## Abbreviations

DSS: dextran sulfate sodium
AOM: azoxymethane
HQD: Huangqin decoction
PCNA: proliferating cell nuclear antigen
MDA: malondialdehyde
MPO: myeloperoxidase
SOD: superoxide dismutase
CRC: colorectal cancer
IBD: inflammatory bowl disease
UC: ulcerative colitis
DAI: disease activity index
H&E: hematoxylin & eosin
PAS: periodic acid-schiff stain
ELISA: enzyme-linked immunosorbent assay
