# Supplementary material for: Protective effects of Huangqin Decoction against ulcerative colitis and associated cancer in mice
Source: Oncotarget. 2016 Aug 19;7(38):61643–55. doi: 10.18632/oncotarget.11426 (PMC5308679; doi:10.18632/oncotarget.11426)
Supplement: Supplementary file 1 [file oncotarget-07-61643-s001.pdf]

Protective effects of Huangqin Decoction against ulcerative colitis and associated cancer in mice

Supplementary Materials

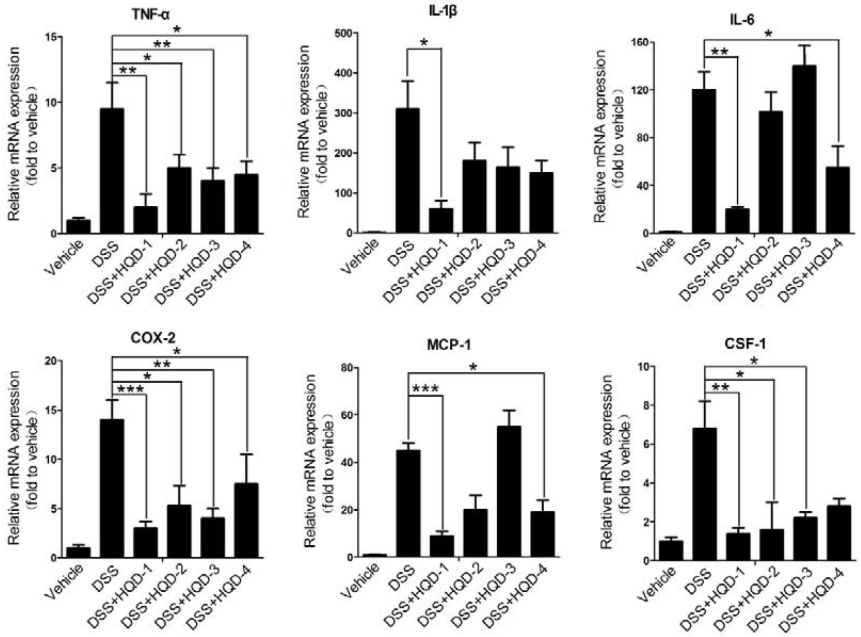

Supplementary Figure S1: Effects of HQD preparations on the production of inflammatory cytokines in colons of mice with DSS-induced acute ulcerative colitis. Quantitative RT-PCR analysis for TNF-α, IL-1β, IL-6, COX-2, MCP-1 and CSF-1 was performed on total RNAs extracted from the colons. All mRNA levels were normalized to the levels of GAPDH mRNA. Data are presented as mean ± SD of 7 mice in each group.**P* < 0.05; ***P* < 0.01; ****P* < 0.001, versus DSS-treated group. HQD, huangqin decoction.

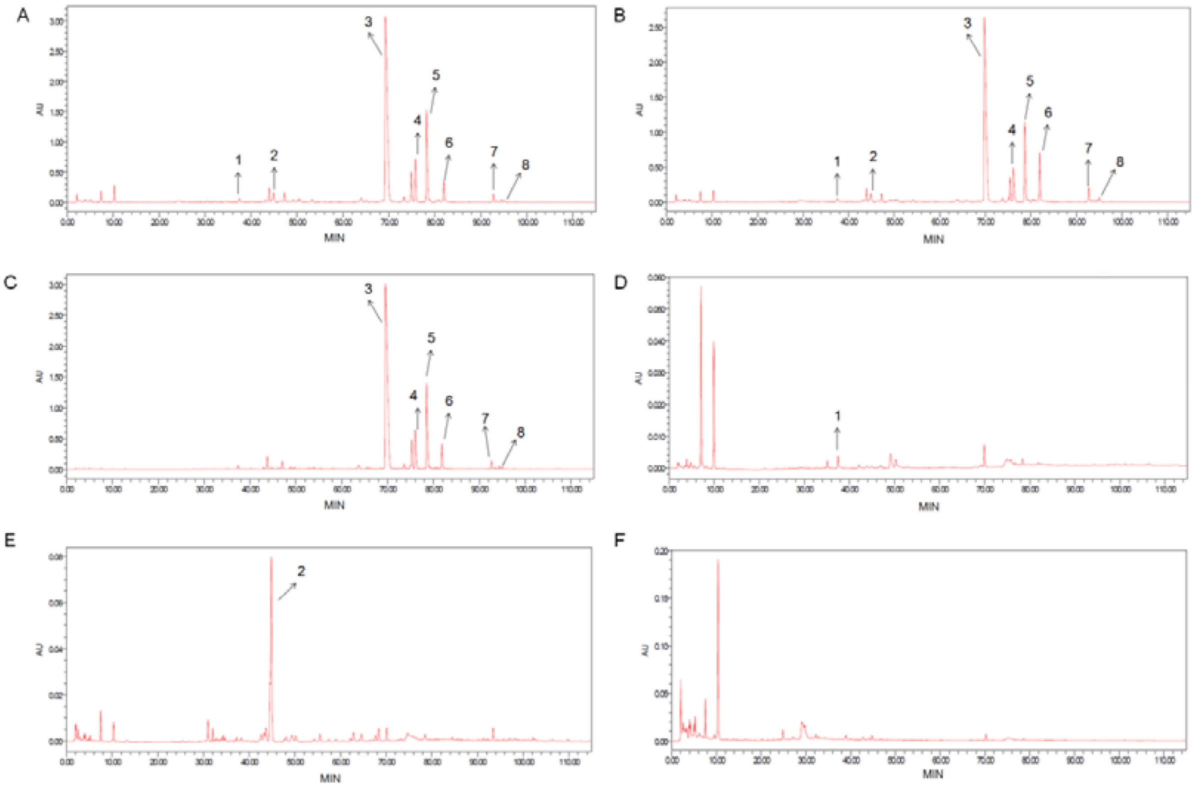

Supplementary Figure S2: HPLC chromatogram of single herbal and HQD extracts. (**A**) HQD-1, (**B**) HQD-2, (**C**) *Scutellaria baicalensis* Georgi, (**D**) *Paeonia lactiflora* Pall, (**E**) *Glycyrrhiza uralensis* Fisch, (**F**) *Ziziphus jujuba* Mill.

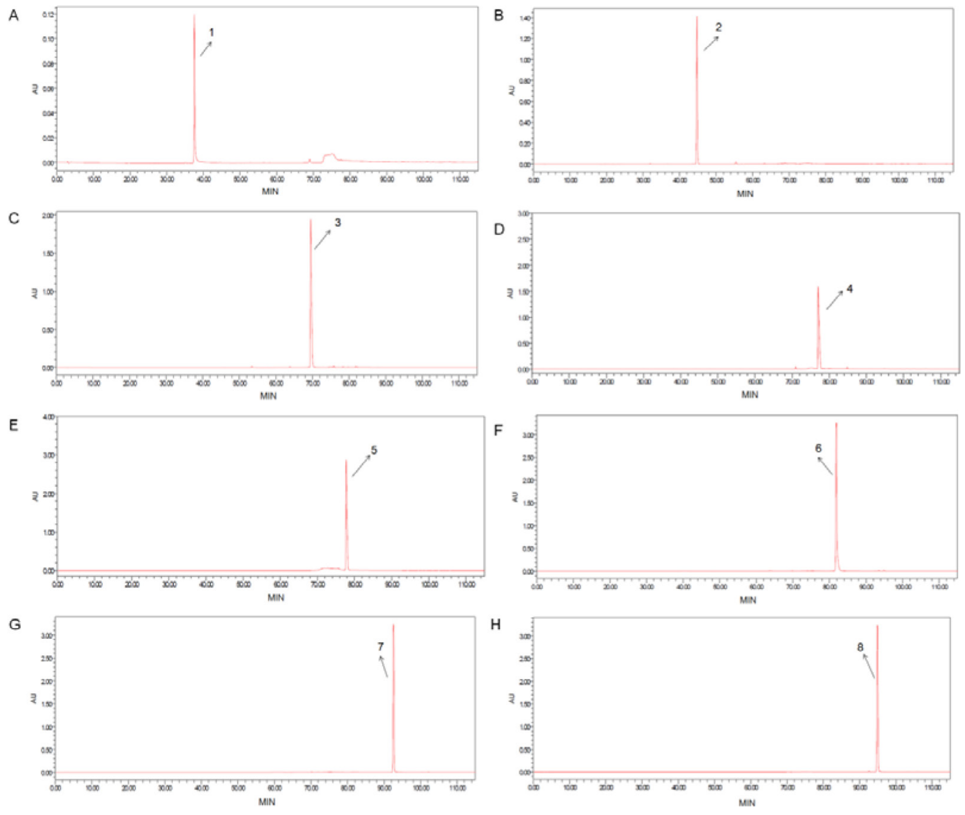

Supplementary Figure S3: HPLC chromatogram of standard. (**A**) Paeoniflorin, (**B**) Liquiritin, (**C**) Baicalin, (**D**) Oroxylin A-7-glucuronide, (**E**) Wogonoside, (**F**) Baicalein, (**G**) Wogonin, (**H**) Oroxylin A.

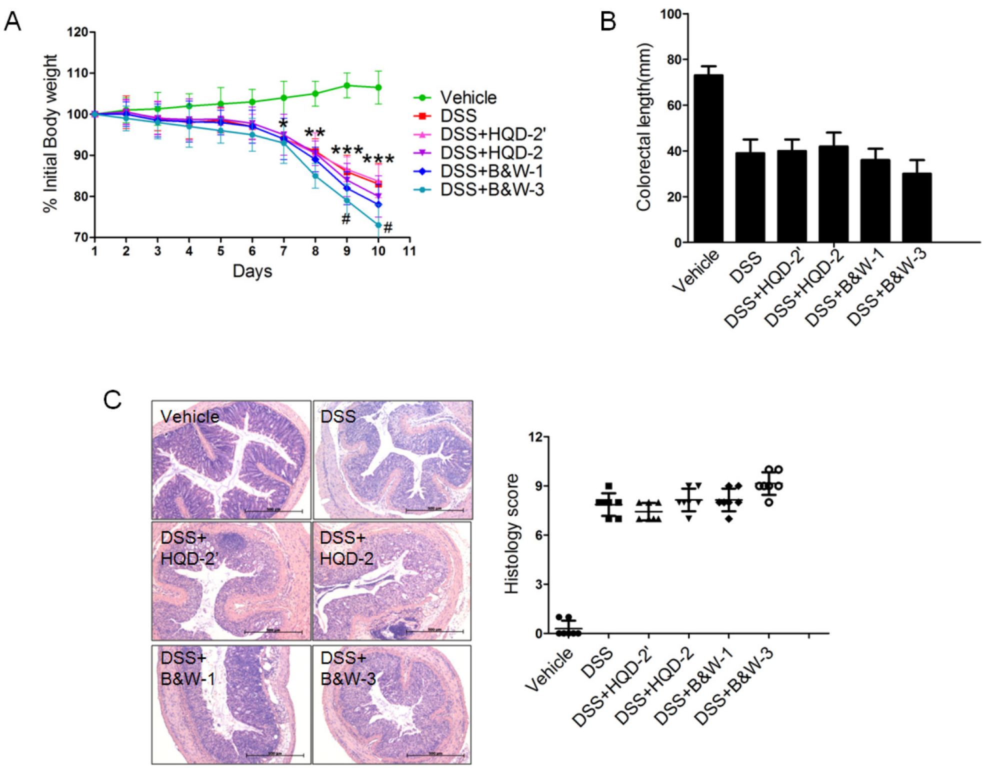

Supplementary Figure S4: The effect of baicalein and wogonin on DSS-induced acute ulcerative colitis in mice. (**A**) Body weight changes after DSS induction of colitis. (**B**) Statistics of colorectum length of each group. (**C**) Representative H&E-stained colorectum sections (magnification×100) and histology score. Data are presented as mean ± SD of 7 mice in each group. *$P < 0.05$; **$P < 0.01$; ***$P < 0.001$, DSS versus vehicle group. $^{\#}P < 0.05$; $^{\#\#}P < 0.01$; $^{\#\#\#}P < 0.001$, DSS+constituents-2 versus DSS group. HQD, huangqin decoction.

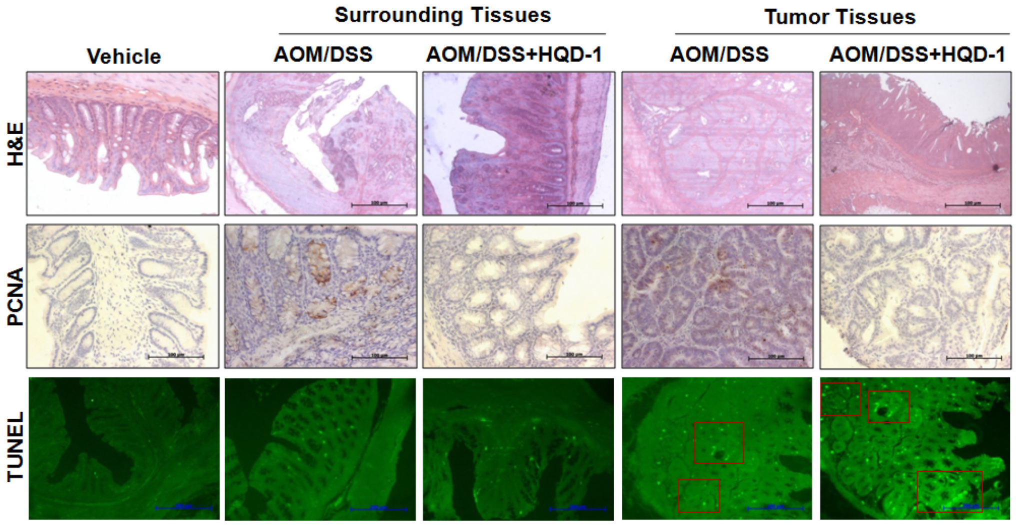

Supplementary Figure S5: HQD-1 suppressed the development of colitis-associated colon cancer. Representative H&E-stained colon sections (magnification ×400), PCNA levels in colonic tissues (magnification ×400) and TUNEL staining in colonic tissues (magnification ×400). HQD, huangqin decoction.

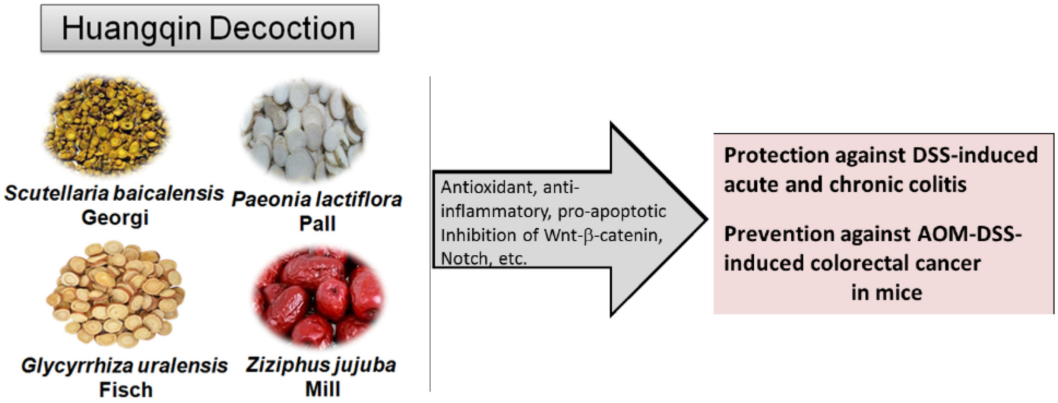

Supplementary Figure S6: The ingredients, activities against colitis and associated colon carcinogenesis, and possible mechanisms of action of Huangqin decoction. The decoction is made from four different herbs. Its prevention against colitis and associated colon carcinogenesis is proposed to be due to the antioxidant, anti-inflammatory and pro-apoptotic activities as well as the inhibition of Wnt-β-catenin, Notch and other molecular pathways.

Supplementary Table S1: The composition of HQD

Species	Chinese name	Plant part	Grams, g
Scutellaria baicalensis Georgi	Huang qin	Root	9
Paeonia lactiflora Pall	Shao yao	Root	6
Glycyrrhiza uralensis Fisch	Gan cao	Root and rhizomes	6
*Ziziphus juju*ba Mill	Da zao	Fruit	49
Total amount			70

Supplementary Table S2: Compounds in HQD

No.	t^R (min)	Identification	Chemical structure	Molecular formula	Source
1	37.561	Paeoniflorin	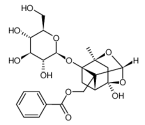	$C_{23}H_{28}O_{11}$	*Paeonia lactiflora* Pall
2	44.797	Liquiritin	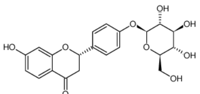	$C_{21}H_{22}O_9$	*Glycyrrhiza uralensis* Fisch
3	54.002	Baicalin	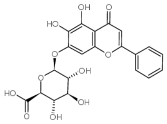	$C_{21}H_{18}O_{11}$	*Scutellaria baicalensis* Georgi
4	75.484	Oroxylin A-7-glucuronide	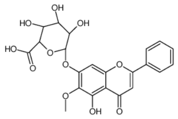	$C_{22}H_{20}O_{11}$	*Scutellaria baicalensis* Georgi
5	78.004	Wogonoside	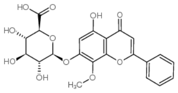	$C_{22}H_{20}O_{11}$	*Scutellaria baicalensis* Georgi
6	81.748	Baicalein	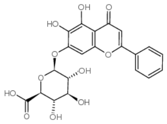	$C_{15}H_{10}O_5$	*Scutellaria baicalensis* Georgi
7	92.537	Wogonin	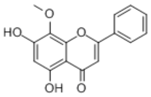	$C_{16}H_{12}O_5$	*Scutellaria baicalensis* Georgi
8	94.824	Oroxylin A	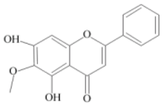	$C_{16}H_{12}O_5$	*Scutellaria baicalensis* Georgi

Supplementary Table S3: Primers used for real-time RT-PCR

Primer	Sequences
TNF-α	Forward primer, 5′- GGAACACGTCGTGGGATAATG
	Reverse primer , 5′- GGCAGACTTTGGATGCTTCTT
IL-1β	Forward primer, 5′- GCAACTGTTCCTGAACTCAACT
	Reverse primer , 5′- ATCTTTTGGGGTCCGTCAACT
IL-6	Forward primer, 5′- TAGTCCTTCCTACCCCAATTTCC
	Reverse primer , 5′- TTGGTCCTTAGCCACTCCTTC
COX-2	Forward primer, 5′- AGGTCATTGGTGGAGAGGTG
	Reverse primer , 5′- CCTGCTTGAGTATGTCGCAC
MCP-1	Forward primer, 5′- AGGTGTCCCAAAGAAGCTGTA
	Reverse primer , 5′- ATGTCTGGACCCATTCCTTCT
CSF-1	Forward primer, 5′- CCCATATTGCGACACCGAA
	Reverse primer , 5′- AAGCAGTAACTGAGCAACGGG
GAPDH	Forward primer, 5′- CTCCCACTCTTCCACCTTCG
	Reverse primer , 5′- CCACCACCCTGTTGCTGTAG

Supplementary Table S4: Validation of linearity, sensitivity, precision, stability and repeatability

**Analyte**	**Linearity**			**Sensitivity (ng/mL)**		**Precision (RSD, %)**		**Stability (RSD,%, *n* = 6)**		**Repeatability (RSD,%, *n* = 3)**	
	**Range (μg/mL)**	**Equation**	**R2**	**LOD**	**LOQ**	**Intra-day (*n* = 6)**	**Inter-day (*n* = 3)**	**HQD-1**	**HQD-2**	**HQD-1**	**HQD-2**
Paeoniflorin (1)	3.815–38.15	$y = 8 \times 10^7x+13445$	$R^2 = 0.990$	863.58	4263.45	1.03	1.29	1.79	0.12	1.29	1.02
Liquiritin (2)	1.155–11.55	$y = 1 \times 10^9x+38914$	$R^2 = 0.993$	67.46	325.50	1.12	2.02	2.98	1.09	1.02	1.79
Baicalin (3)	1.21–12.1	$y = 1 \times 10x+3 \times 106$	$R^2 = 0.997$	360	3025	2.15	2.15	2.02	2.19	2.15	2.13
Oroxylin A-7-glucuronide (4)	2.125–34.0	$y = 3 \times 10^9x+3 \times 106$	$R^2 = 0.991$	474.3	1517.85	2.79	2.09	2.14	2.42	2.32	2.92
Wogonoside (5)	4.25–34.0	$y = 3 \times 10^9x-7 \times 106$	$R^2 = 0.991$	2547.72	2421.84	2.23	1.29	1.23	1.18	1.23	1.09
Baicalein (6)	0.745–7.45	$y = 5 \times 10^9x-20357$	$R^2 = 0.999$	21.88	72.53	1.28	1.02	1.22	0.98	1.22	0.98
Wogonin (7)	0.113–2.25	$y = 1 \times 10^{10}x+17890$	$R^2 = 0.999$	5.82	31.66	2.79	1.79	1.79	0.77	0.79	0.79
